# Supplementary material for: Interventions to Foster Resilience in Family Caregivers of People with Alzheimer’s Disease: A Scoping Review
Source: Int J Environ Res Public Health. 2024 Apr 16;21(4):485. doi: 10.3390/ijerph21040485 (PMC11050178; doi:10.3390/ijerph21040485)
Supplement: Supplementary file 1 [file ijerph-21-00485-s001.zip › ijerph-2924711-supplementary.pdf]

Supplementary Material

Interventions to Foster Resilience in Family Caregivers of People with Alzheimer's Disease: A Scoping Review

Table S1. Qualitative studies (methodological assessment following the recommendations of the CASP guide).

**Intervention**	**Author Year**	**Was there a clear statement of the aims of the research?**	**Is a qualitative methodology appropriate? the research?**	**Was the research design appropriate to address the aims of the research?**	**Was the recruitment strategy appropriate to the aims of the research?**	**Was the data collected in a way that addressed the research issue?**	**Has the relationship between researcher and participants been adequately considered?**	**Have ethical issues been taken into consideration?**	**Was the data analysis sufficiently rigorous?**	**Is there a clear statement of findings?**	**How valuable is the research?**
Creative Art	Pienaar L. 2015	*	*	*	*	*	*	*	*	*	*
Creative Art	Hunt B. 2016	*	*	*	*	*	*	*		*	*

Table S2. Mixed studies (methodological assessment by the MMAT tool).

Intervention	Author Year	Clear question	Appropriately collected data	Adequate justification	Integrated components to answer the research question	Appropriate interpretation of the integration of qualitative and quantitative	Adequate addressing of the inconsistencies of both types of studies	Quality criteria for both studies separately
Creative Art	Kidd L. 2011	*	*	*	*	*		*
Creative Art	McMAnus 2021	*	*	*	*			

Table S3. Experimental and quasi-experimental studies (methodological assessment following the CASP tool).

Intervention	Author Year	Did the study address a clearly focused research question?	Was the assignment of participants to Interventions Randomised?	Were all participants who entered the study accounted for at its conclusion?	were the participants, ‘ blind’ to intervention they were given?	Were the study groups similar at the start of the randomised controlled trial?	Apart from the experimental intervention, did each study group receive the same level of care (that is, were they treated equally)?	Were the effects of intervention reported comprehensively?	Was the precision of the estimate of the intervention or treatment effect reported?	Do the benefits of the experimental intervention outweigh the harms and costs?	Can the results be applied to your local population/in your context?	Would the experimental intervention provide greater value to the people in your care than any of the existing interventions?
Meditation program	Pandya SP 2019	*	*	*	*	*	*	*	*		*	
Transcendental Meditation	Leach MJ 2015	*	*	*	*	*	*		*	*		
Psychoeducational program	Orrell M 2017	*	*	*	*	*		*	*		*	
Psychoeducational program	Cerquera A 2017	*	*							*	*	
Psychoeducational program	Ghaffari F 2019	*		*		*	*		*	*		*
